# Supplementary material for: Tribological Properties of Nitrate Graphite Foils
Source: Nanomaterials (Basel). 2024 Sep 15;14(18):1499. doi: 10.3390/nano14181499 (PMC11434555; doi:10.3390/nano14181499)
Supplement: Supplementary file 1 [file nanomaterials-14-01499-s001.zip › X-ray data/misorientation/01Hz/over500/dens_10/RD/137.pdf]

Raw data

Sample Name:
File : 137.raw
Comment :
Date : May-06-24 12:43:51
Operator : Administrator

X-ray : Cu / 40 kV / 30 mA
Goniometer : Ultima IV
Attachment : MPA-ML4(with gamma) for stress
Filter : K-beta filter
I.Monochro : CBO
C.Monochro : Not installed
DivSlit : Open
DivH.L.Slit : 10.00mm
SctSlit : 2/3?‹|2/3deg.
RecSlit : 0.30mm

Counter : Scintillation counter
Scan mode : Continuous
Scan speed : 5.00 deg./min.
Sampling width : 0.05 no unit
Scan axis : theta
Scan range : 10.00 -> 80.00 no unit
Fixed angle : 0.00 no unit

Memo :

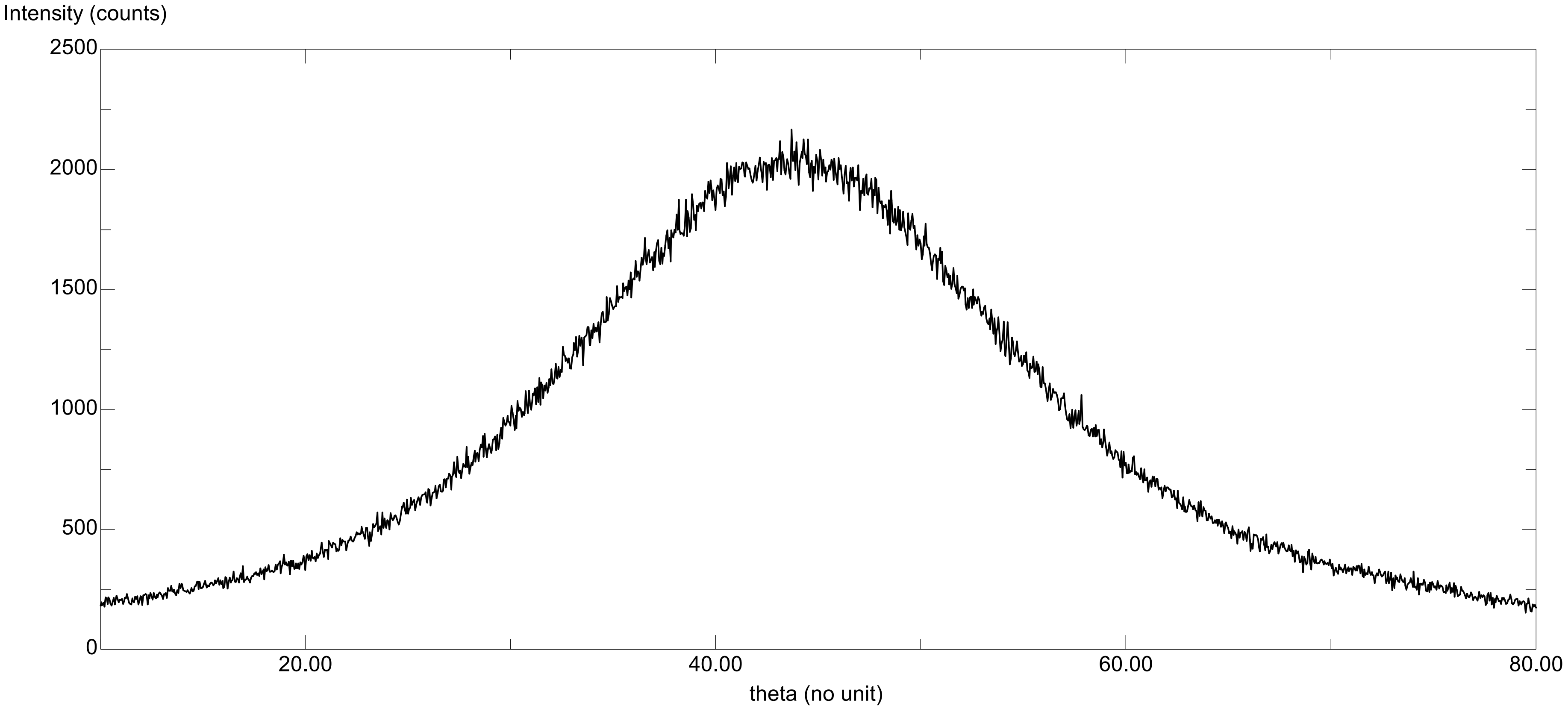

Integral Int. Calculation (History)

Sample Name: File : 137.raw Date : May-06-24 12:43:51 Operator : Administrator
Comment : Memo :
Peak calc. method: FW2/3M

h	k	l	Start	Stop	Peak	d-value	FWHM	Max. int.		Gross int.	Integ.w	Integ. int.
0	0	0	10.00	no unit80.00	no unit43.85	no unit2.06	24.33	no unit1841	counts	988898.63	26.861	49444.93

[Cut]
[Slit Correction]
[LP Corr.]

[Absorp. Corr.]
[Norm.]
[Smoothing] Method : Averaging Smoothing Points: 11
[BG subtraction] Method : Averaging at the end
Low angle : 5 High angle : 12
[Ka2 elimination]

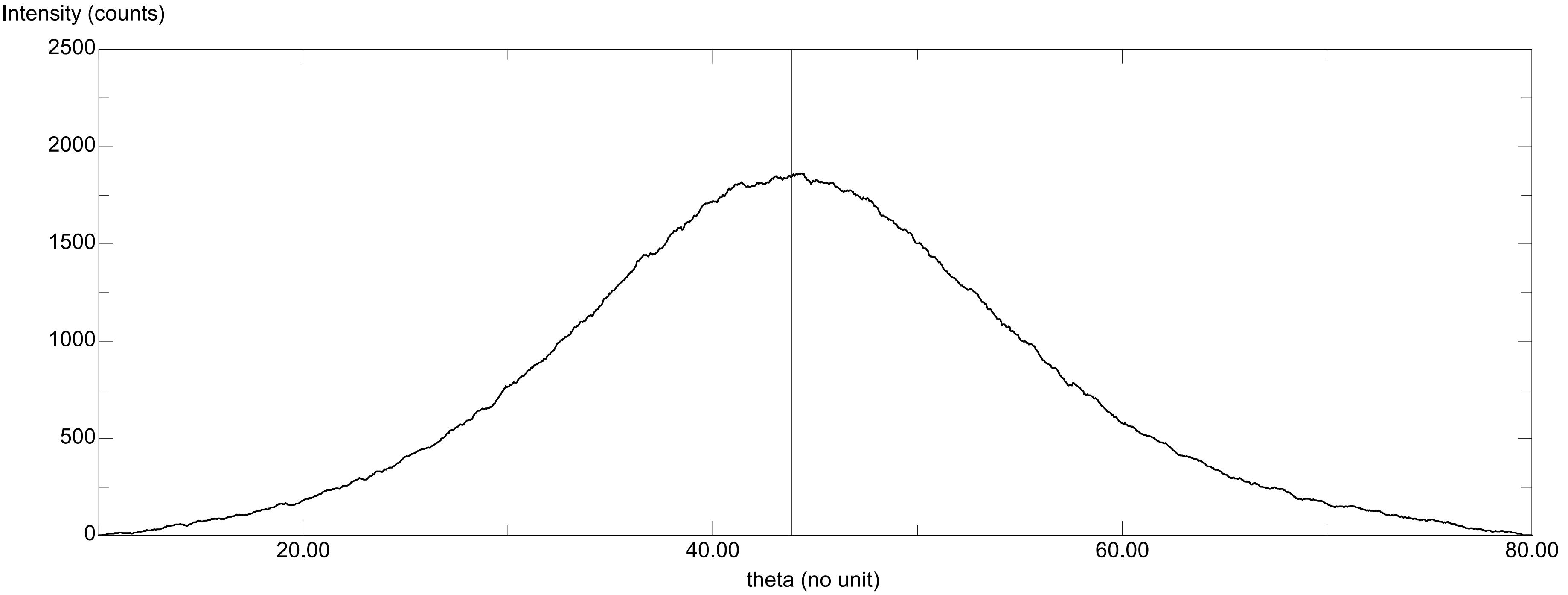

Integral int. sum table

Sample Name: File : 137.raw Date : May-06-24 12:43:51 Operator : Administrator
Memo :
Peak calc. method: FW2/3M

No.	h	k	l	Start	Stop	Peak	d-value	FWHM	Max. int.	Gross int.	Integ.w	Integ. int.	Comment
1	0	0	0	10.00	80.00	43.85	2.06	24.33	1841	988898.63	26.861	49444.93	
